# A new genus and species of Sphyrapodidae (Crustacea, Peracarida, Tanaidacea) from the southern coast of the South Korea

**DOI:** 10.3897/zookeys.735.14671

**Published:** 2018-02-06

**Authors:** Jin Hee Wi, Chang-Keun Kang

**Affiliations:** 1 School of Earth Sciences and Environmental Engineering, Gwangju Institute of Science and Technology, Gwangju 61005, Republic of Korea

**Keywords:** Tanaidacea, Apseudomorpha, Sphyrapodinae, *Wandogarida*, sexual dimorphism

## Abstract

*Wandogarida canalicula* **gen. n et sp. n.** (Tanaidacea, Sphyrapodidae) is described from the southern coast of Korea (NW Pacific). The genus is included in the subfamily Sphyrapodinae *Guţu*, 1980 by having a mandible lacking a palp. It has a close affinity with the genus *Poligarida* Bamber & Marshall, 2013 in the third pereonite having lateral pointed apophyses, the antennule of males with fringes of aesthetascs on articles 1 and 2 of the outer flagellum and the antenna with an elongate article 2. However, it is distinguished from *Poligarida* by having a maxillular palp. *Wandogarida* **gen. n.** can be differentiated from other genera within the subfamily Sphyrapodinae by the unique shape characterised by the male’s antennule article 1 with its rough denticles and a deep groove, pleonites each with a ventral hyposphenium, the maxillule with uniarticulate palp and the presence of prominent sexual dimorphism in the mouthparts. An identification key to the five genera of the subfamily Sphyrapodinae is presented.

## Introduction

The suborder Apseudomorpha Sieg, 1980 includes about 460 species and is widely distributed in various shallow marine benthic habitats, being quite abundant in coral reefs, estuaries and mangrove swamps, from the tropics to temperate regions (Błażewicz-Paszkowycz and Bamber 2012). Although many species of this suborder have been described from diverse regions, the current state of the systematics is unstable and unresolved (Larsen 2012). Guţu (1980) established the family Sphyrapidae, with two subfamilies Pseudosphyrapinae Guţu, 1980 and Sphyrapinae Guţu, 1980, to encompass a group of tanaidaceans characterised by the enlarged pereopod 1, the lack of spiniform apophyses on the carapace, the biramous antennule, the mandible with or without palp and the maxillule with palp. Later their familial and subfamilial names were emended to Sphyrapodidae Guţu, 1980 and Sphyrapodinae Guţu, 1980 and Pseudosphyrapodinae Guţu, 1980 by Larsen (2005). The family has undergone several systematic reviews to establish its current taxonomic status (see Anderson 2013). Initially, the family Sphyrapodidae had been placed in the superfamily Metapseudoidea Guţu, 1981 within the suborder Monokonophora Lang, 1956 (as Sphyrapidae). However, the suborder and superfamily were not accepted and Metapseudoidea was synonymised with Apseudoidea Leach, 1814 by Sieg (1984).

More than 300 sphyrapodid specimens were collected during a recent survey of the shallow mud sandy seabed from the southern coast of South Korea. Their examination revealed the presence of a new species in a new genus belonging to the subfamily Sphyrapodinae.

## Materials and methods

The materials were obtained from the sandy bottom off Wando Island on the southern coast of South Korea: (34°31.1'N; 128°33.2'E at a depth of 41 m) in October 2015 using an epi-sledge net. The specimens were extracted by filtering the substrates through a 350 μm sieve and the residue from each sieve was preserved in a 99% alcohol solution. Later, the animals were identified and counted in the laboratory. The specimens were dissected under a dissection microscope (Nikon SMZ745T) in CMC-10 aqueous mounting medium (Masters, Wood Dale, IL, USA), mounted on slides and then sealed with high-quality nail varnish. Drawings were generated using a differential interference contrast microscope (Nikon Y-IM) that was equipped with a drawing tube. The total body length was measured from the tip of the rostrum to the pleotelson apex in the dorsal view. Scale bars are given in mm. The morphological terminology follows Larsen (2003). The type and other materials examined were deposited in the Marine Biodiversity Institute of Korea (MABIK), Seocheon, South Korea.

## Systematics

### Order Tanaidacea Dana, 1849

#### Suborder Apseudomorpha Sieg, 1980

##### Superfamily Apseudoidea Leach, 1814

###### Family Sphyrapodidae Guţu, 1980

####### Subfamily Sphyrapodinae Guţu, 1980

######## Genus Wandogarida gen. n.

Taxon classificationAnimaliaTanaidaceaSphyrapodidae

http://zoobank.org/C75030C0-3F54-4156-B4BD-00A5D666E67F

Figs 1 , 2 , 3 , 4 , 5 , 6 , 7 , 8 , 9

######### Generic diagnosis.

Rostrum narrow and prominently extended. Carapace wider than long. Pereonite 3 with lateral apophyses. Pleonites each with a ventral spur and pointed epimera. Pleotelson with slight distal extension. Antennule inner flagellum biarticulate; peduncle article 1 in males with a vertical row of rough denticles and groove. Antenna 8-articulate, without squama. Mandible without palp; molar with distal setulose setae and spinose cutting edge. Maxillule with uniarticulate palp. Pereopods 2–4 propodus with ventral seta. Uropod exopod 3-articulate.

######### Etymology.

The name refers to Wando, a port city near the type locality and *garida* from the Greek γαίδα, meaning “shrimp” (feminine).

######### Type species.

*Wandogarida canalicula* sp. n.

######### Remarks.

*Wandogarida* gen. n. is classified in the subfamily Sphyrapodinae following Guţu (1980), Larsen (2005) and Bamber and Marshall (2013), with a definition based on the following morphological features: 1) the rostrum is prominently extended anteriorly; 2) the pereonites are all wider than long; 3) the antennule has a short inner flagellum with 1–2 articles; 4) the mandible is without a palp; 5) the maxillule is with or without a palp; and 6) the pereopod 1 and cheliped are with exopod.

The subfamily is now composed of five genera: *Ansphyrapus* Guţu, 2001, *Poligarida* Bamber & Marshall, 2013, *Sphyrapoides* Guţu & Iliffe, 1998 and *Sphyrapus* Sars, 1882, including the new genus *Wandogarida*.

*Wandogarida* resembles *Poligarida* in the absence of an antennal squama, antennule with a biarticulate inner flagellum, pereonite 3 with anterolateral pointed apophyses and outer flagellum of the male antennule with fringes of aesthetascs. However, *Wandogarida* can be differentiated from *Poligarida* by the following: in both sexes, the number of antenna articles is different (8 vs. 7); the maxillule has a uniarticulate palp (vs. absence); the carpus and propodus of pereopods 2–3 and propodus of the pereopod 4 have ventral spiniform setae (vs. absence); in females, the mandible molar has several setulose distal setae and sharp, spinose distal margin (vs. with distal setae and simple distal edge); in males, sexual dimorphism exists in the antennule article 1 with a vertical row of rough denticles and concave distolateral margin, in reduced and simplified mandibles, maxillule, maxilla and maxilliped endite, in the larger and more robust cheliped and in the shape of ventral margin of the pereopod 1 dactylus, while it exists only in the antennule, cheliped and pereopod 1in *Poligarida*.

### Key to the genera of the subfamily Sphyrapodinae

**Table:** 

1	Antennule inner flagellum with 0–1 articles	***Sphyrapus***
–	Antennule inner flagellum with 1–2 articles	**2**
2	Antenna with squama	***Sphyrapoides***
–	Antenna without squama	**3**
3	Maxillule with palp	**4**
–	Maxillule without palp	***Wandogarida* gen. n.**
4	Pereopod 6 with many long setae along basis, merus and carpus	***Ansphyrapus***
–	Pereopod 6 without many long setae along basis, merus and carpus	***Poligarida***

#### Wandogarida canalicula sp. n.

Taxon classificationAnimaliaORDOFAMILIA

http://zoobank.org/BBEE7CE0-F2C4-42A4-ABD6-DD3D12A836EE

Figs 1 , 2 , 3 , 4 , 5 , 6 , 7 , 8 , 9

##### Type-specimens.

**Holotype**: (MABIK CR00240685) female dissected and mounted on five slides. **Allotype**: (MABIK CR00240686) male dissected and mounted on five slides; from the same locality as the holotype. **Paratypes**: Four females partly dissected on one slide (MABIK CR00240687) and in 3 vials (MABIKCR00235359–MABIKCR00235361); from the same locality as the holotype. Four males partly dissected on one slide (MABIK CR00240688) and in 2 vials (MABIKCR00235362, MABIKCR00235363); from the same locality as for holotype.

##### Type-locality.

Wando, South Korea (North West Pacific), 34°31.02'N, 128°33.11'E, mud-sandy bottom at a depth of 41 m.

##### Etymology.

The specific name is derived from Latin *canalicula*, meaning a groove and refers to the conspicuous groove formed on the distal margin of the male antennule.

##### Descriptions.

**Figure 1.**
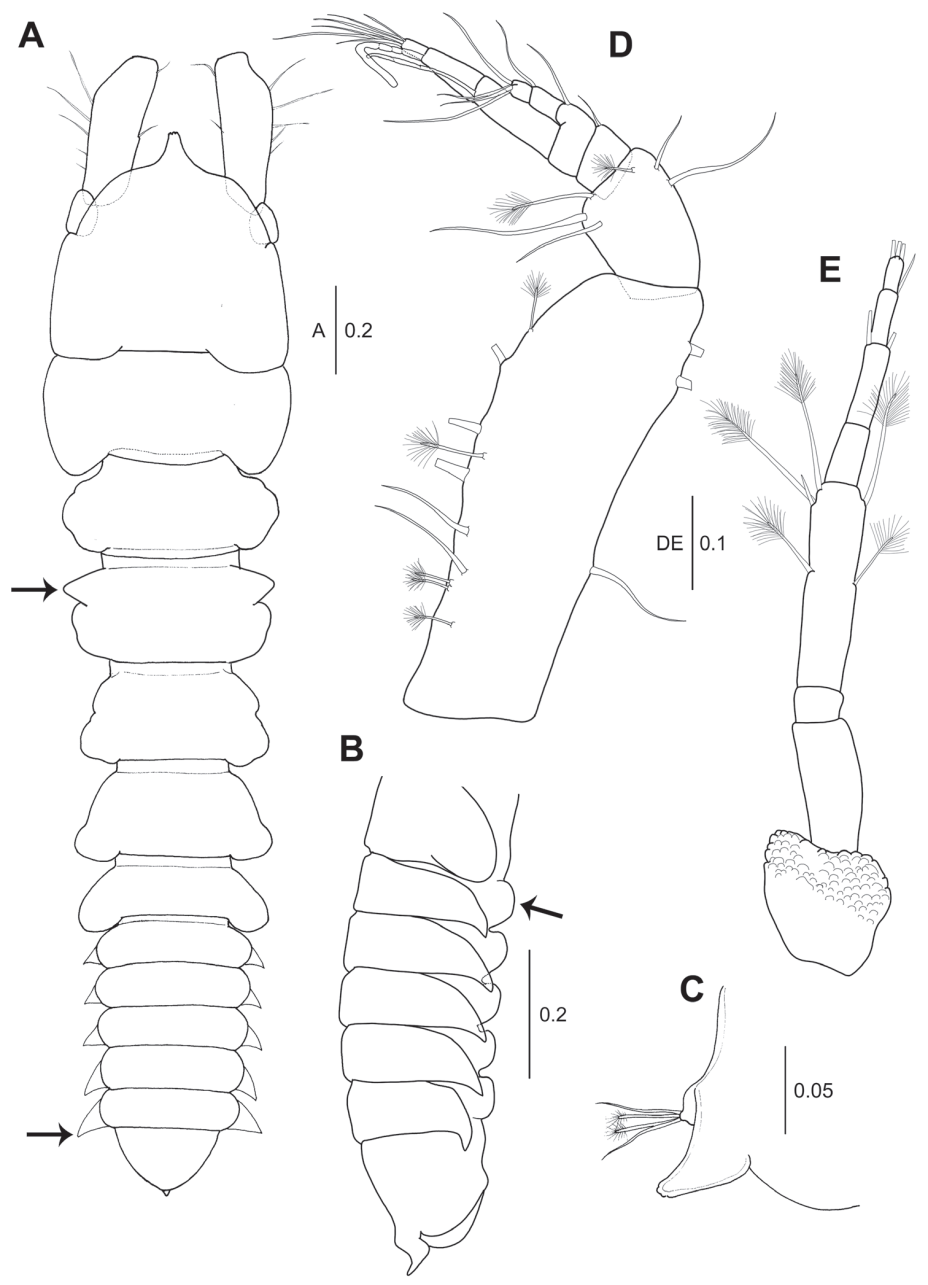
*Wandogarida canalicula* gen. n., sp. n., holotype, female: **A** habitus, dorsal view, upper arrow indicating apophysis of pereonite 3 and lower arrow indicating epimera of pleonite 5 **B** pleon, lateral view, arrow indicating hyposphenium of pleonite 1 **C** pleotelson, lateral view **D** antennule **E** antenna. Scale bars are given in mm.

***Female* (with oostegites).** Body (Fig. 1A), dorsoventrally flattened, holotype 2.4 mm long, 4.3 times as long as wide. Cephalothorax 20.6 % of body length, slightly wider than long, gradually widening posteriorly, proximal margin with conspicuous extended rostrum with or without small protrusions. Eyes well developed, without pigmentation. Pereonites each with different shape. Pleonites each with pointed epimera. Pleotelson gradually tapered.

*Pereon* (Fig. 1A): 53 % of body length. Pereonite 1 not fused to cephalothorax, 0.6 times as long as cephalothorax, half as long as wide, posterolateral margins rounded and distally extended. Pereonite 2 medially swollen, slightly shorter than pereonite 1, 0.4 times as long as wide. Pereonite 3 subequal length of pereonite 2, half as long as wide, anterolateral margin with apophysis (arrowed), mid-lateral margin swollen. Pereonite 4 subtrapezoidal, as long as pereonite 2, 0.6 times as long as wide, mid to posterolateral margin gradually widening. Pereonite 5 as long as pereonite 4, half as long as wide, posterolateral margin rounded and slightly extended. Pereonite 6 shortest, 0.4 times as long as wide, posterolateral margin rounded and extended.

*Pleon* (Fig. 1A, B): 20.2 % of body length, 1.1 times as long as wide, pleonites each with pointed epimera, of which pleonite 5 is largest (arrowed) and rounded ventral hyposphenium (arrowed in Fig. 1B). Pleotelson (Fig. 1A–C): distally tapered, 6.6 % of total length, distally with two broom setae and two simple setae of almost equal length (Fig. 1C).

*Antennule* (Fig. 1D): Peduncle article 1 robust, 57 % of total length, 2.9 times as long as wide, with three simple setae on inner margin and five simple setae and five broom setae on outer margin. Article 2 0.3 times as long as article1, 1.5 times as long as wide, with two simple setae and one broom seta each on outer and inner distal margin. Article 3 0.3 times as long as article 2, with one simple inner distal seta. Article 4 slightly shorter than article 3, with one simple inner distal seta. Inner flagellum biarticulate: article 1 with one distal simple seta and article 2 with four distal simple setae. Outer flagellum 3-articulate, each article gradually shortened: article 1 with one aesthetasc; article 2 with one aesthetasc and; article 3 shortest, with six distal simple setae.

*Antenna* (Fig. 1E): 8-articulate, slender and shorter than antennule. Article 1 distally wider and covered with scale-like ornamentations. Articles 2, 3 and 5 naked. Article 4 longest, outer margin each with one broom seta medially and distally, inner margin with one medial and two distal broom setae and one distal simple seta. Article 6 with two distal simple setae. Article 7 with one distal simple seta. Article 8 as long as article 3, with three distal simple setae. Proportional lengths of articles 16.8: 18.0: 5.1: 27.9: 8.4: 11.3: 7.6: 4.9.

**Figure 2.**
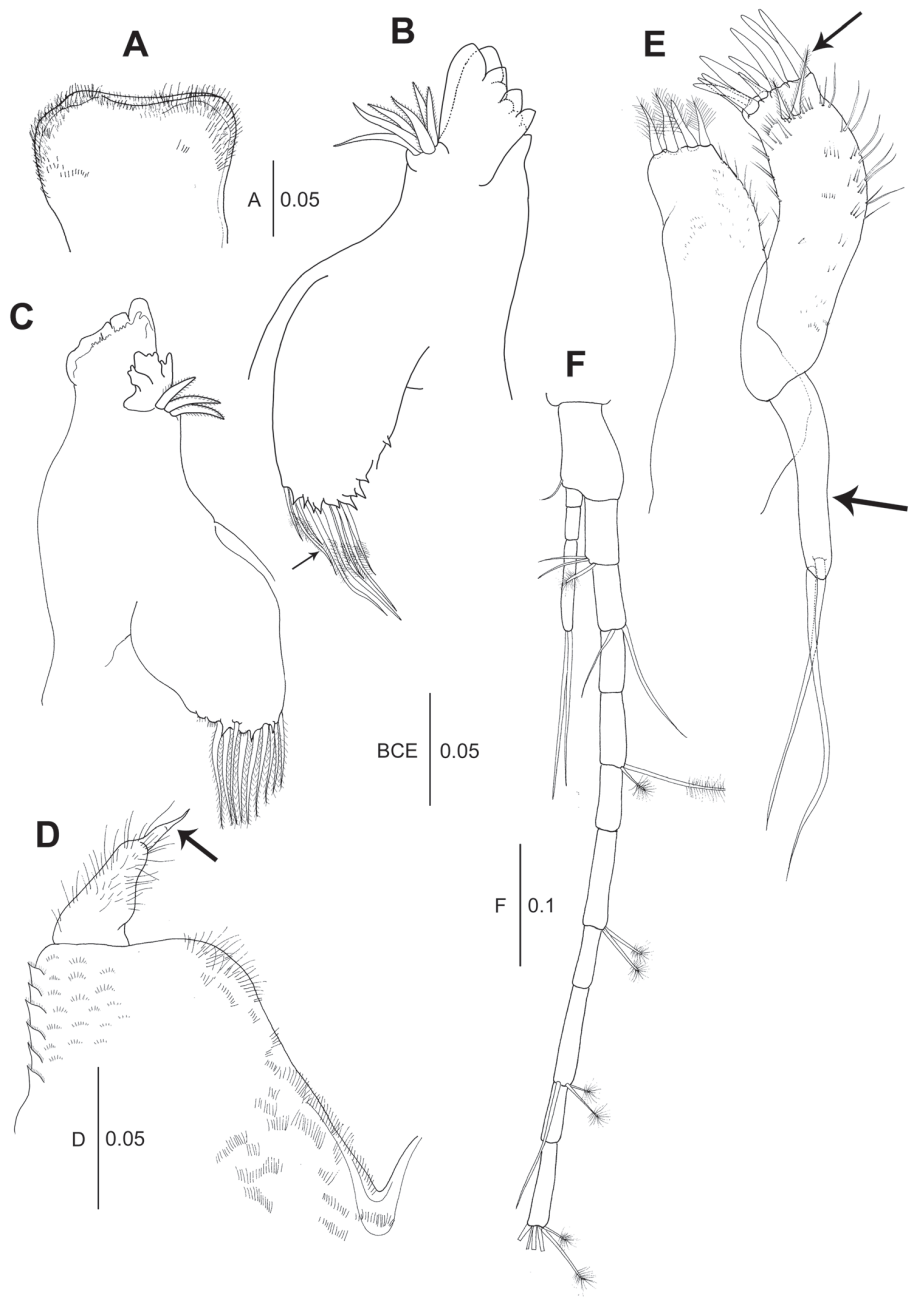
*Wandogarida canalicula* gen. n., sp. n., holotype, female: **A** labrum **B** left mandible, arrow indicating eight distal setulose setae on molar **C** right mandible **D** labium, arrow indicating distal spine of palp **E** maxillule, upper arrow indicating two subdistal setulose spiniform setae on outer endite and lower arrow indicating uniarticulate palp **F** uropod. Scale bars are given in mm.

*Labrum* (Fig. 2A): Sub-rectangular, distal margin covered with numerous setules.

*Left mandible* (Fig. 2B): Incisor with six prominent denticles distally; lacinia mobilis with four distal denticles; setal row with six setulose spiniform setae; molar distally tapered, with sharp, spinose distal edge and eight long setulose setae (arrowed). *Right mandible* (Fig. 2C) incisor with three irregular distal denticles; setal row with one tripartite seta, which is substantially larger than others and three setulose spiniform setae; lacinia mobilis absent; molar similar to that of left mandible. Palp absent.

*Labium* (Fig. 2D): Lobe with setulate spines and microtrichia along outer margin and ornamented with setules along inner margin. Palp covered with setules and one distal spine (arrowed).

*Maxillule* (Fig. 2E): Inner endite with four setulose setae on distal margin; outer margin ornamented with somewhat long setules. Outer endite with nine distal spiniform setae, two subdistal setulose spiniform setae and setules. Palp uniarticulate (arrowed), with one subterminal and one terminal simple setae.

**Figure 3.**
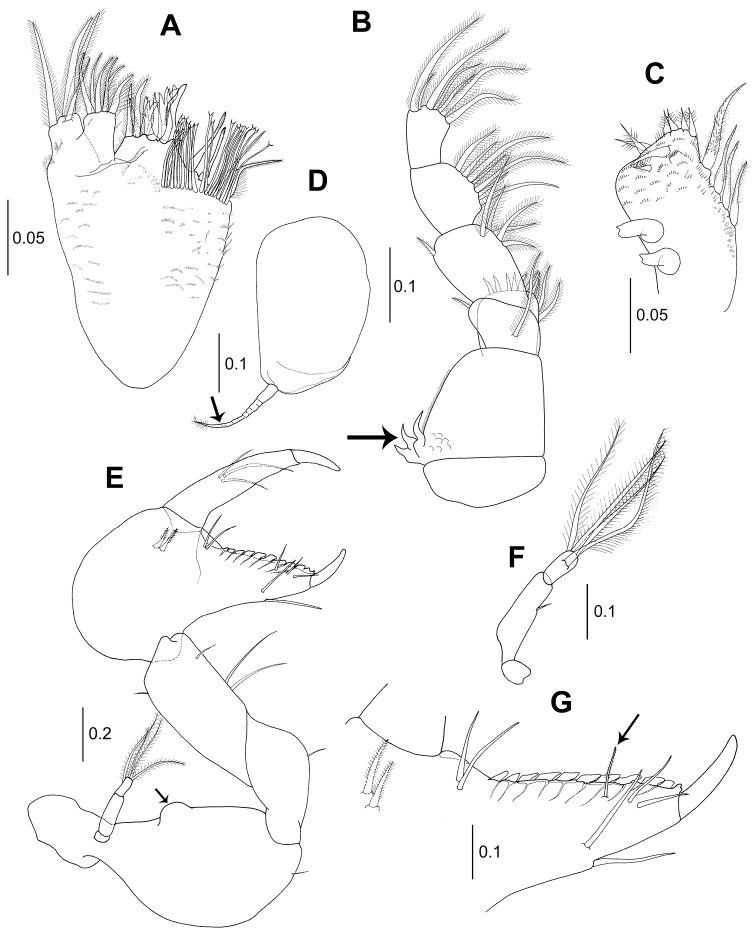
*Wandogarida canalicula* gen. n., sp. n., holotype, female: **A** maxilla **B** maxilliped, arrow indicating three curved spines on basis **C** maxilliped endite **D** epignath, arrow indicating long wrinkled seta on distal margin **E** cheliped, arrow indicating dorsomedial protrusion on basis **F** cheliped exopodite **G** fixed finger of cheliped, arrow indicating setulose seta near cutting edge. Scale bars are given in mm.

*Maxilla* (Fig. 3A): Outer lobe of movable endite with four setulose setae. Inner lobe of movable endite with six setulose setae. Outer lobe of fixed endite with six setulose spiniform setae and four setulose tripartite spiniform setae. Inner lobe of fixed endite with four setulose spiniform setae on distal margin and 20 simple bifid tipped setae.

*Maxilliped* (Fig. 3B, C): Coxa naked. Basis with three curved robust spines on outer proximal margin (arrowed). Palp article 1 with one short seta on outer distal margin and one subdistal setulose seta on medial margin. Article 2 longer than wide, with one distal setulose seta on outer margin and five subdistal setae on inner margin. Article 3 with seven setulose setae along inner margin. Article 4 with seven distal setulose setae. Endite (Fig. 3C) with four stout setulose spiniform setae on inner margin, two coupling hooks on outer margin, six setulose spiniform setae on distal margin, two setulose spiniform setae on subdistal margin and several microtrichia. Epignath (Fig. 3D) rounded, with long distally setulose and wrinkled seta on distal margin (arrowed).

*Cheliped* (Fig. 3E–G): Basis rounded and with dorsomedial protrusion (arrowed), 1.9 times as long as wide, with one ventrodistal simple seta; exopod 3-articulate, with four plumose setae on distal margin (Fig. 3F). Merus ventrally rounded, shorter than basis, with ventromedial simple seta. Carpus sub-rectangular, with three simple setae on ventral margin and one subdistal simple seta on dorsal margin. Propodus longer than basis, merus and carpus, with two simple setae and two setulose spiniform setae near insertion of dactylus. Fixed finger with ten blunt denticles along cutting edge, one setulose seta (arrowed in Fig. 3G) and two simple setae on inner margin and two simple setae on ventral margin. Dactylus with three simple setae on inner medial margin, cutting edge smooth.

**Figure 4.**
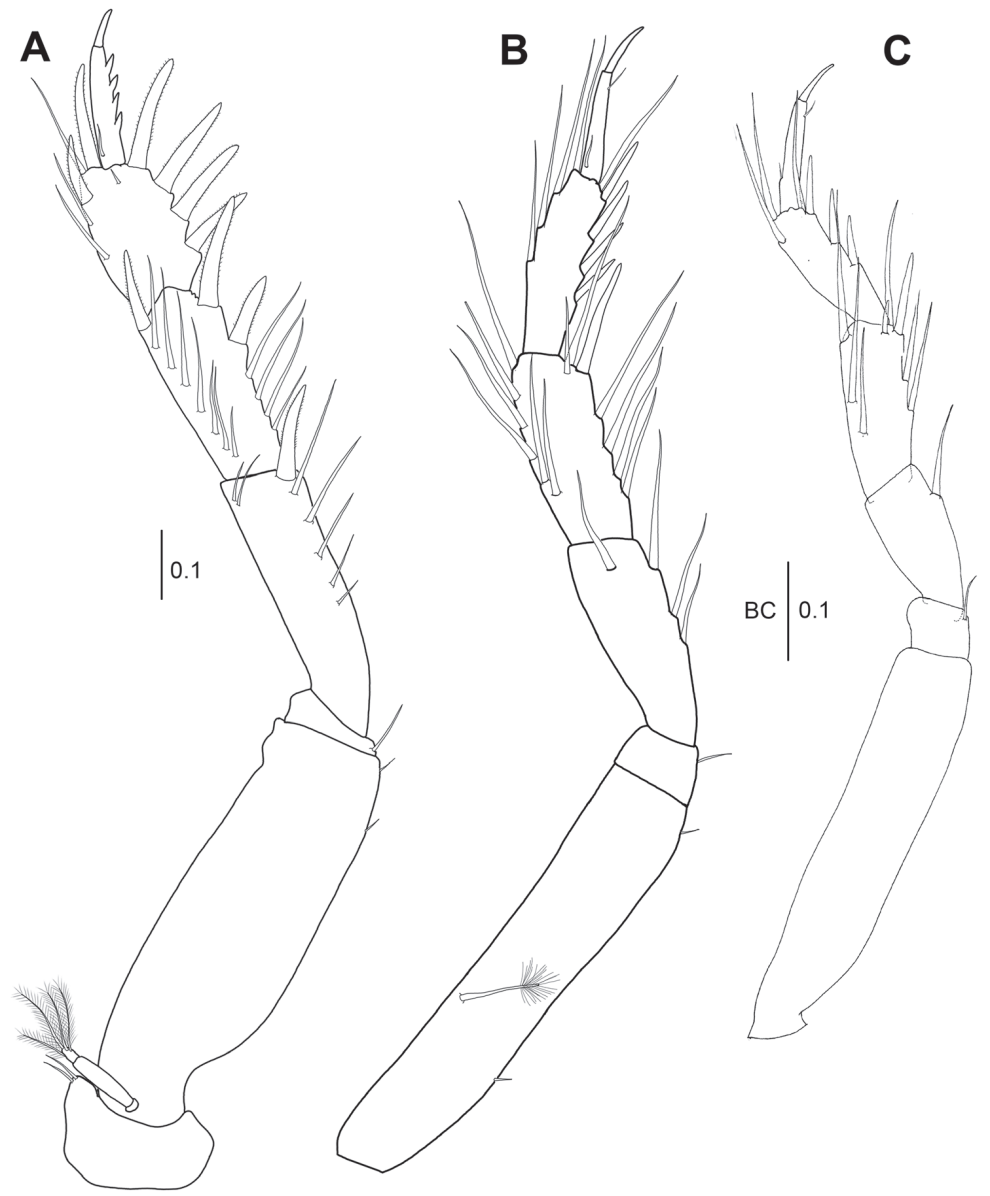
*Wandogarida canalicula* gen. n., sp. n., holotype, female: **A** pereopod 1 **B** pereopod 2 **C** pereopod 3. Scale bars are given in mm.

*Pereopod 1* (Fig. 4A): Larger than pereopods 2–6, spiniform setae ornamented with small setules bilaterally. Coxa with two dorsodistal simple setae. Basis robust, three times as long as wide, with two short simple setae on ventrodistally. Exopod 3-articulate, distal article with four distal plumose setae. Ischium compact, with one simple ventrodistal seta. Merus 0.6 times as long as basis, 3.3 times as long as wide, with five ventral simple setae and one ventrodistal spiniform seta and two dorsodistal simple setae. Carpus 0.8 times as long as merus, 2.3 times as long as wide, ventral margin with four slender simple setae and two setulose spiniform setae and dorsally with seven simple slender setae and one setulose spiniform seta. Propodus with four strong spiniform setae on ventral margin, one short subdistal simple seta and two distal spiniform setae and three simple setae along dorsal margin. Dactylus and unguis combined subequal to propodus. Dactylus stout, with four pointed denticles along ventral margin and one simple slender seta on subproximal margin; unguis 0.3 times as long as dactylus.

*Pereopod 2* (Fig. 4B): Basis 4.8 times as long as wide, with two ventral short simple setae and one dorsal broom seta. Ischium with one ventrodistal simple seta. Merus with three simple ventral setae and one subdistal simple seta. Carpus slightly shorter than merus, with six simple setae and one spiniform seta on ventral margin and six simple setae along dorsal margin. Propodus slightly shorter than merus, with four spiniform setae and one distal simple seta along ventral margin and four simple setae along dorsal margin. Dactylus and unguis combined 0.8 times as long as propodus, with one dorsoproximal simple seta and one ventrodistal simple seta. Unguis 0.4 times as long as dactylus.

*Pereopod 3* (Fig. 4C): Basis shorter than that of pereopod 2, 4.8 times as long as wide, with one ventrodistal simple seta. Ischium with one ventrodistal simple seta. Merus 0.3 times as long as basis, with one ventro-subdistal simple seta. Carpus 1.2 times as long as merus, with three simple setae and two spiniform setae of unequal length on ventral margin and three simple setae along dorsal margin. Propodus as long as merus, with two simple setae on dorsodistal margin and three spiniform setae and one distal simple seta on ventral margin. Dactylus and unguis combined 1.2 times as long as propodus. Unguis 0.4 times as long as dactylus.

**Figure 5.**
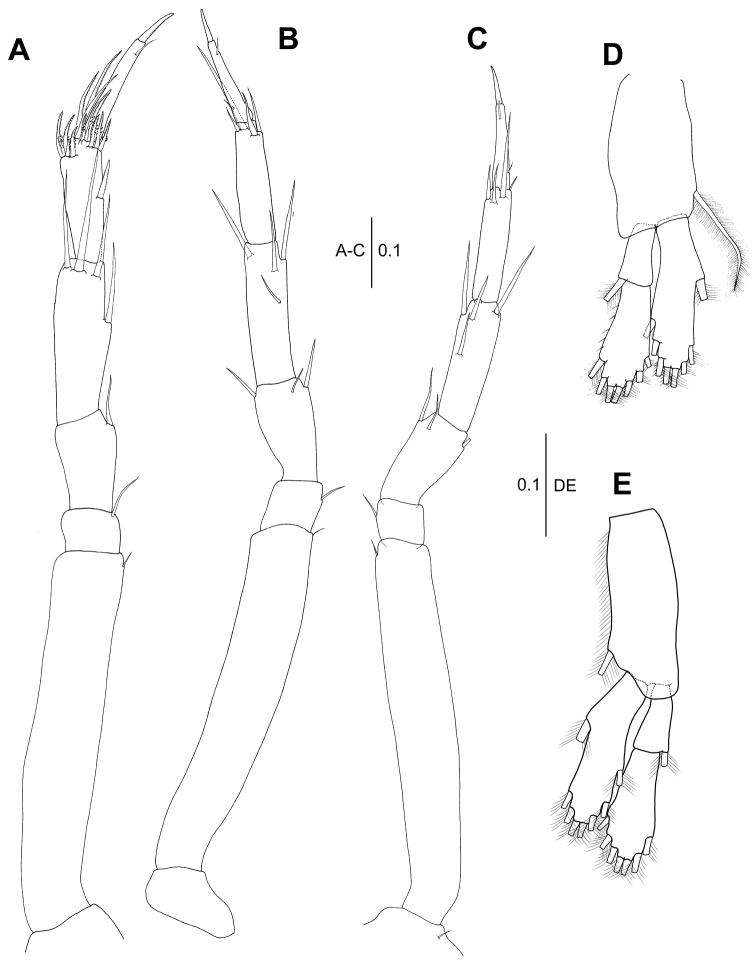
*Wandogarida canalicula* gen. n., sp. n., holotype, female: **A** pereopod 4 **B** pereopod 5 **C** pereopod 6 **D** pleopod **E** male, pleopod. Scale bars are given in mm.

*Pereopod 4* (Fig. 5A): Basis subequal length of pereopod 2, 5.5 times as long as wide, with one simple ventrodistal seta. Ischium with one simple ventrodistal seta. Merus 0.2 times as long as basis, 1.6 times as long as wide, with one simple seta on ventro-subdistal margin. Carpus 1.6 times as long as merus, 2.7 times as long as wide, with one midventral simple seta and three distal simple setae. Propodus 0.8 times as long as carpus, with one setulose spiniform seta on midventral margin and twelve serrated setae on distal margin. Dactylus and unguis combined 1.3 times as long as propodus. Unguis 0.4 times as long as dactylus.

*Pereopod 5* (Fig. 5B): Basis shorter than that of pereopod 2, 5.6 times as long as wide. Ischium similar to that of pereopod 4. Merus as long as pereopod 4, 0.3 times as long as basis, 1.8 times as long as wide, with one distal simple seta each on ventral and dorsal margin. Carpus 1.5 times as long as merus, 3.6 times as long as wide, with three simple setae on distal and subdistal margins. Propodus 0.8 times as long as carpus, 3.6 times as long as wide, with four simple setae on distal margin. Dactylus and unguis subequal length of propodus. Unguis similar to that of pereopod 4.

*Pereopod 6* (Fig. 5C): Basis 4.4 times as long as wide. Ischium like that of pereopod 5. Merus 0.3 times as long as basis, 1.8 times as long as wide, with three subdistal setae. Carpus 1.3 times as long as merus, with three simple setae subdistally and one simple seta medially. Propodus 0.9 times as long as carpus, with four simple setae on distal margin. Dactylus and unguis 1.2 times as long as propodus. Unguis 0.3 times as long as dactylus.

*Pleopods 1–5* (Fig. 5D): Alike. Biramous. Basal article 1.9 times as long as wide, with one plumose seta on inner distal margin. Endopod shorter than exopod, with one plumose seta on inner medial margin and seven distal and outer plumose setae. Endopod biarticulate, article 1 with one outer distal plumose seta; article 2 with seven distal plumose setae.

*Uropod* (Fig. 2F): Basal article 1.5 times as long as wide, with simple seta on distal margin. Exopod 3-articulate, article 3 1.7 times as long as articles 1 and 2 combined, with two plumose setae on tip of article 3. Endopod 10-articulate, distal article with four simple setae and two broom setae.

**Figure 6.**
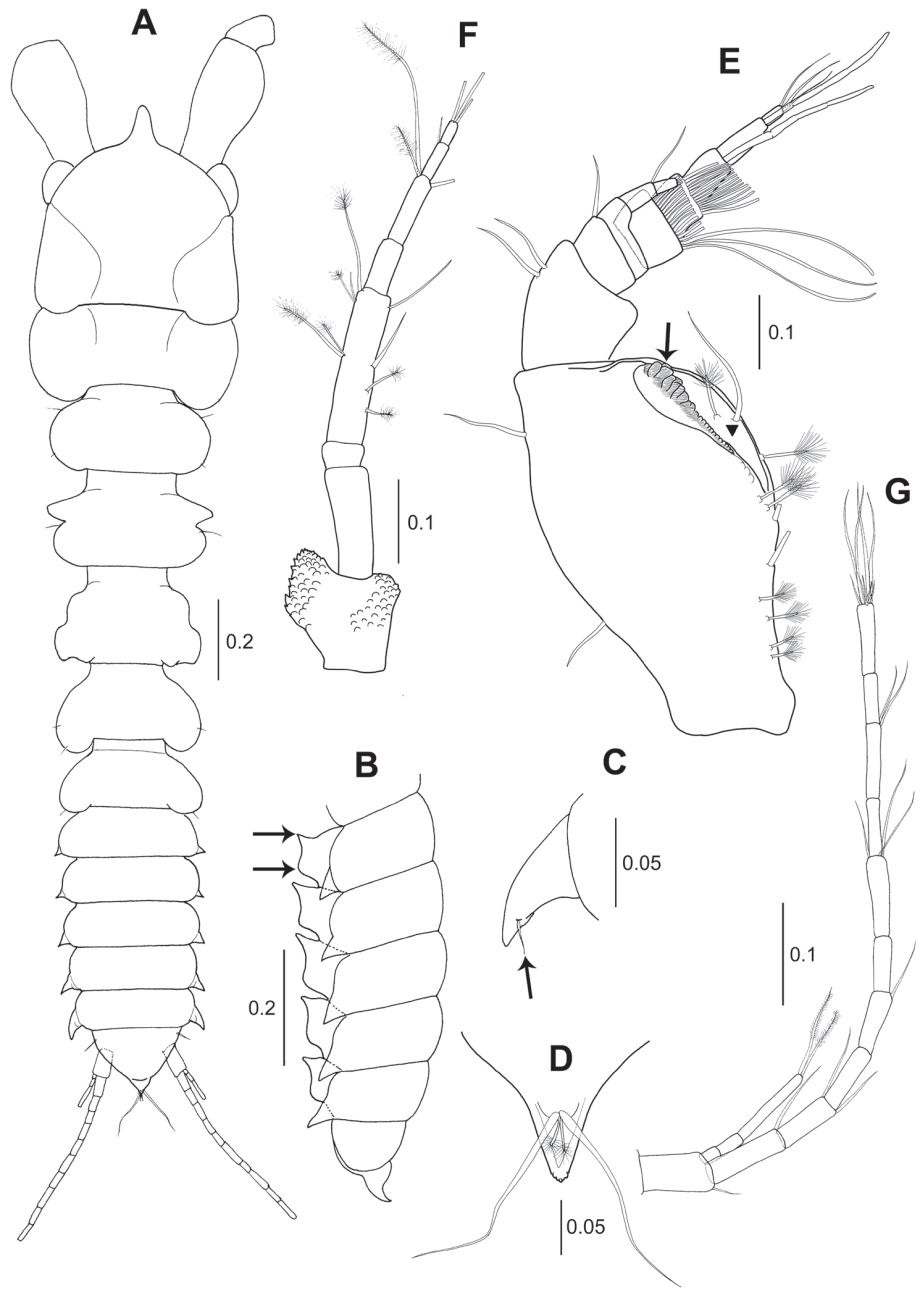
*Wandogarida canalicula* gen. n., sp. n., allotype, male: **A** habitus, dorsal view **B** pleonites and pleotelson, lateral view, arrows indicating upper and lower margins of hyposphenium of pleonite 1 **C** epimera of pleonite, arrow indicating simple seta **D** distal extension of pleotelson, ventral view **E** antennule, arrow indicating projection having row of rough denticles and inverted triangle indicating deep groove between projection and smooth margin **F** antenna **G** uropod. Scale bars are given in mm.

***Male*.** Body (Fig. 6A), dorsoventrally flattened, 2.4 mm long, 4.7 times as long as wide, pereonites each with different shape. Cephalothorax 20.5 % of body length, as long as wide, anterior margin with conspicuous elongate, rounded rostrum. Eyes well developed, without pigmentation.

*Pereon* (Fig. 6A): Wider than long, 50.3 % of body length. Pereonite 1 0.4 times as long as cephalothorax, 0.4 times as long as wide, lateral margin rounded and posteriorly extended. Pereonite 2 laterally rounded, 1.15 times as long as pereonite 1, half as long as wide. Pereonite 3 slightly longer than pereonite 2, 0.6 times as long as wide, anterolateral margin with pointed apophysis. Pereonite 4 subequal length of pereonite 3, 0.7 times as long as wide. Pereonite 5 0.8 times as long as pereonite 4, half as long as wide, posterolateral margin rounded and posterior margin extended. Pereonite 6 shortest of all pereonites, half as long as wide.

*Pleon* (Fig. 6A–D): 22 % of body length. Pleonites subequal in length, each with anteriorly pointed and posteriorly rounded ventral hyposphenium (arrowed in Fig. 6B). Epimera pointed and gradually becoming larger posteriorly, with one simple seta (arrowed in Fig. 6C). Pleotelson (Fig. 6A, B, D): 7 % of body length, as long as pereonite 6, with two simple setae on lateral margin, two broom setae and two long simple setae of unequal length on posterior margin.

*Antennule* (Fig. 6E): Peduncle article 1 longer and more robust than other articles, outer distal margin with projection having row of rough denticles (arrowed) and mid to distal surface with deep groove between projection and smooth margin (marked by inverted triangle), 47 % of total length, 1.7 times as long as wide, with eight broom setae and three simple setae on outer margin and two simple setae on inner margin. Article 2 about 0.4 times as long as article 1, with two simple setae on inner margin. Article 3 about 0.4 times as long as article 2, 0.6 times as long as wide, with one inner distal simple seta. Article 4 shorter than article 3, naked. Inner flagellum biarticulate: article 1 with inner distal simple seta and article 2 with three distal simple setae. Outer flagellum 5-articulate: articles 1 and 2 wider than long, with fringes of aesthetascs distally; article 3 with one aesthetasc; article 4 longest, with one aesthetasc; article 5 shortest, with three simple setae.

*Antenna* (Fig. 6F): Similar to those of female. Proportional lengths of articles 15.7: 19.3: 4.2: 26.6: 9.1: 14.6: 6.3: 4.2.

*Labrum* and *labium* almost equal to those of female.

**Figure 7.**
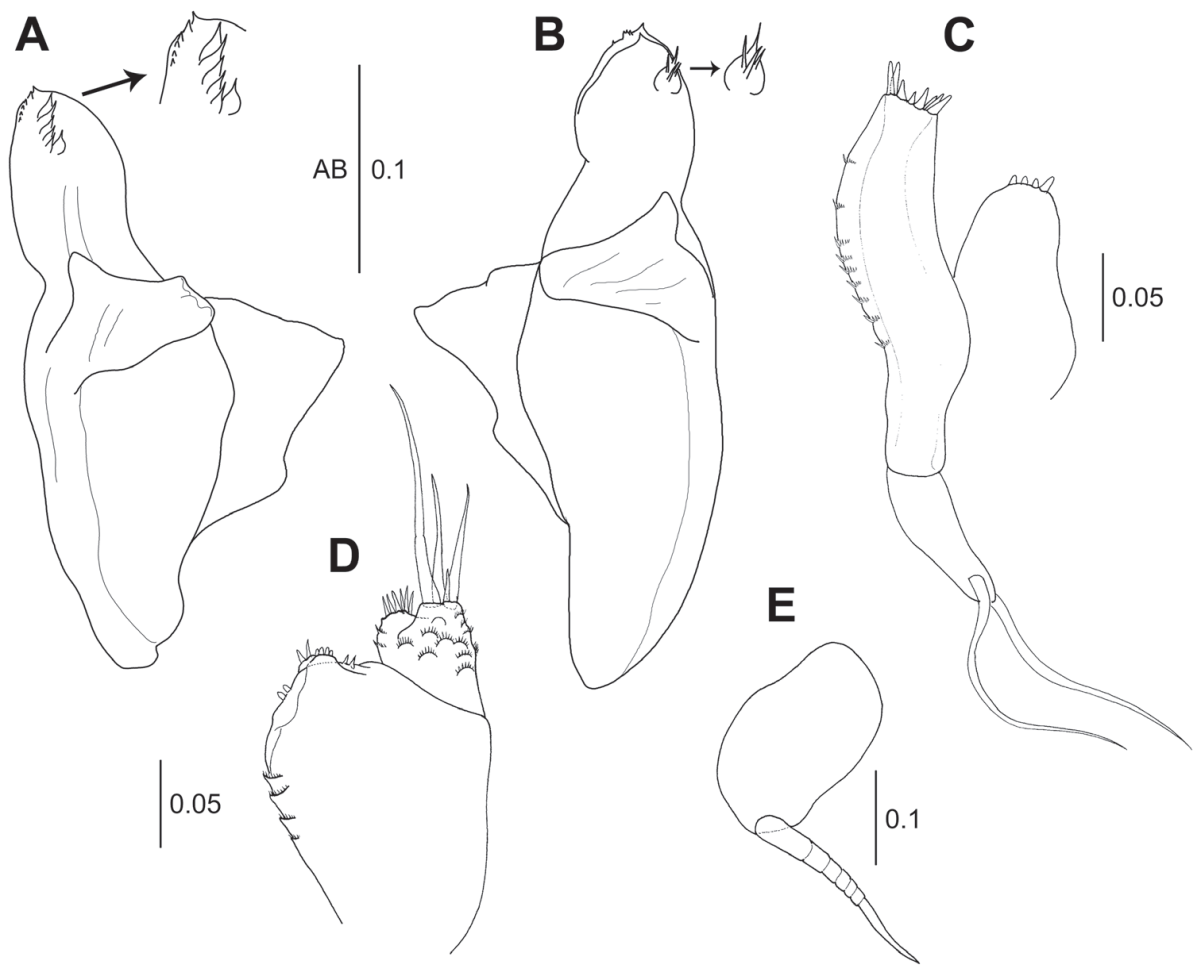
*Wandogarida canalicula* gen. n., sp. n., allotype, male: **A** right mandible **B** left mandible **C** maxillule **D** maxilla **E** epignath. Scale bars are given in mm.

*Left mandible* (Fig. 7B): Incisor terminally pointed, with small denticles. Lacinia mobilis distally pointed. Setal row with four small setae (arrowed). Molar subtriangular and reduced compared to female. *Right mandible* (Fig. 7A): incisor with distal denticles. Setal row with vertical row of denticles and one pointed seta. Molar similar to that of left mandible.

*Maxillule* (Fig. 7C): Reduced and simplified compared to that of female. Inner endite with four short simple spiniform setae. Outer endite with eight distal simple spiniform setae. Palp with one subdistal and one distal simple setae.

*Maxilla* (Fig. 7D): Movable endite with microtrichia on surface: outer lobe with four simple setae; inner lobe with six short simple setae. Fixed endite reduced and simplified: outer lobe with seven short simple setae; inner lobe very reduced, with two short simple setae.

**Figure 8.**
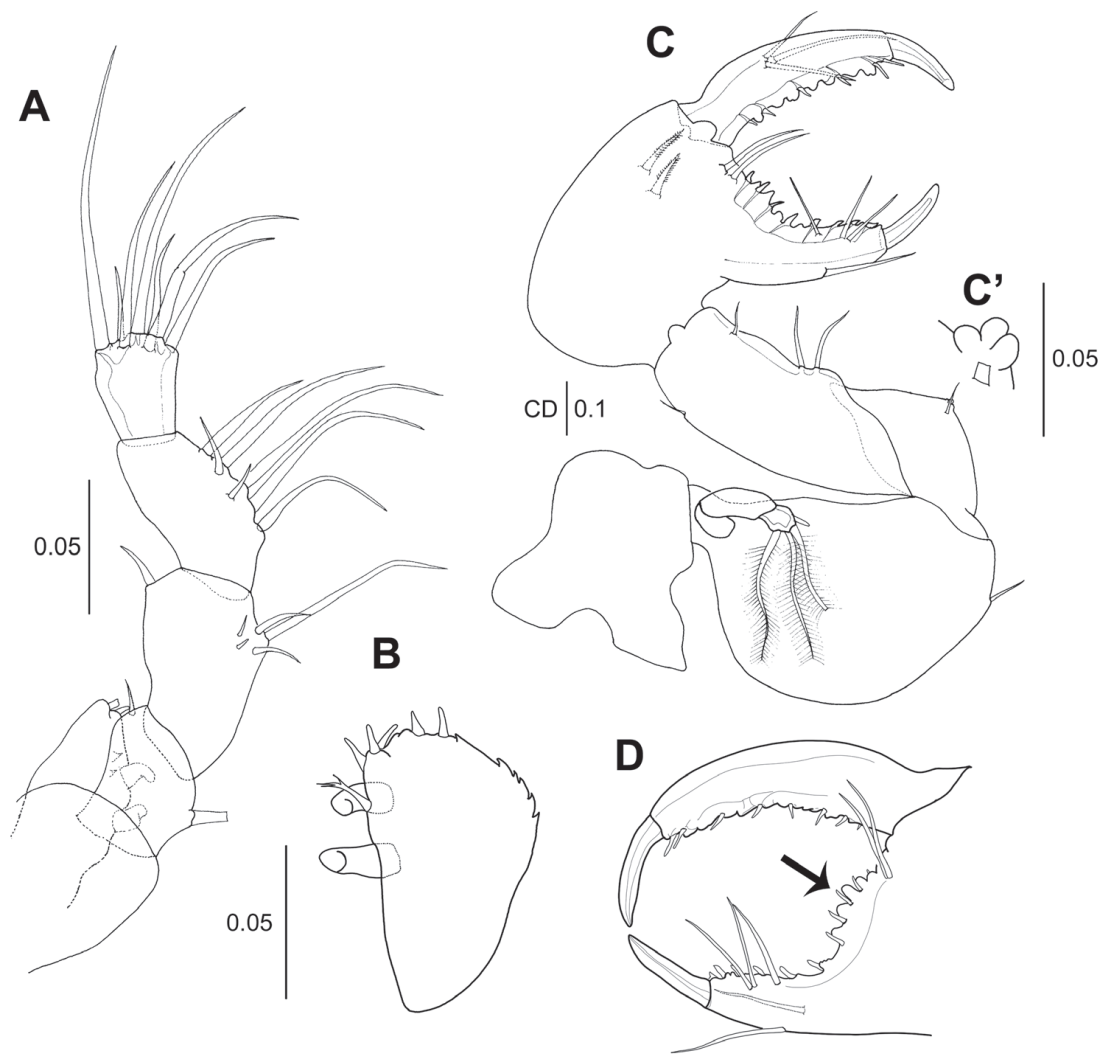
*Wandogarida canalicula* gen. n. sp. n., allotype, male: **A** maxilliped **B** maxilliped endite **C** cheliped **C**’ processes on the cheliped merus **D** cheliped, fixed finger and dactylus, arrow indicating denticle on cutting edge. Scale bars are given in mm.

*Maxilliped* (Fig. 8A, B): Palp articles similar to those of female. Endite (Fig. 8B) simplified: distal and subdistal margins with six short simple spiniform setae, inner margin with two coupling hooks and outer margin with several spines. *Epignath* (Fig. 7E): Smaller than that of female, distal seta longer than lobe.

*Cheliped* (Fig. 8C, C’, D): Basis rounded, 1.5 times as long as wide, with one ventrodistal seta; exopodite similar to that of female. Merus with one simple seta and three processes on midventral margin (enlarged in Fig. 8C’). Carpus similar to that of female. Propodus 2.5 times as long as carpus, setation equal to that of female. Fixed finger with one ventral and one medial simple setae, cutting edge with three simple setae and several denticles and blunt processes. Dactylus as long as fixed finger, with three simple setae on inner medial margin and several blunt processes and denticles along cutting edge.

**Figure 9.**
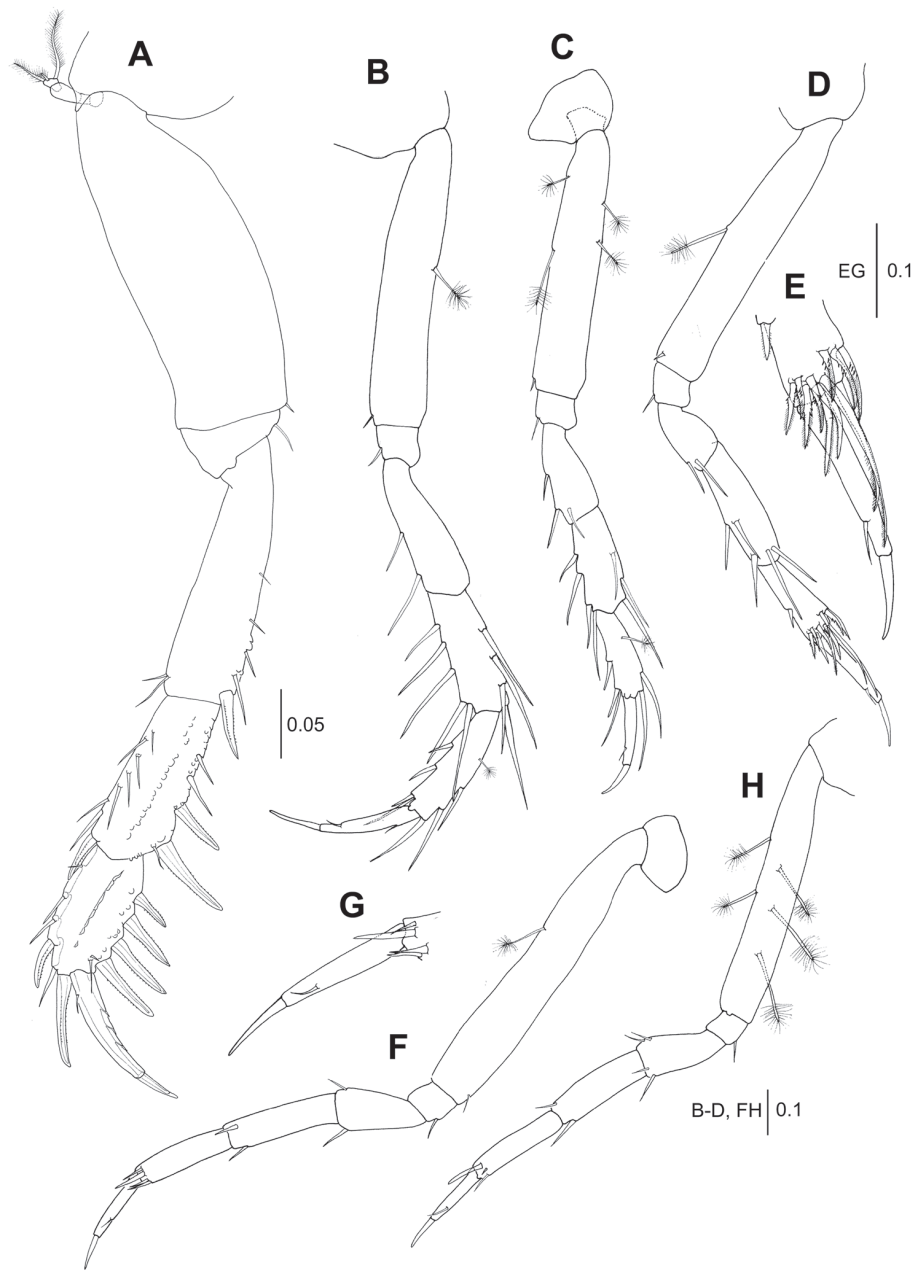
*Wandogarida canalicula* gen. n., sp. n., allotype, male: **A** pereopod 1 **B** pereopod 2 **C** pereopod 3 **D–E** paratype, male **D** pereopod 4 **E** pereopod 4, distal margin of propodus, dactylus and unguis **F–H** allotype, male **F** pereopod 5 **G** pereopod 5, dactylus and unguis **H** pereopod 6. Scale bars are given in mm.

*Pereopod 1* (Fig. 9A): Basis 2.7 times as long as wide, with one ventrodistal simple seta. Ischium with one ventrodistal simple seta. Merus 0.7 times as long as basis, 2.3 times as long as wide, with two dorsodistal simple setae, one ventrodistal spiniform seta and four ventral simple setae. Carpus 0.7 times as long as merus, 2.3 times as long as wide, with two ventrodistal spiniform setae, three ventral simple setae, one dorsodistal spiniform seta and seven dorsal simple setae. Propodus 0.7 times as long as carpus, 1.8 times as long as wide, with four ventral spiniform setae, two ventral simple setae near insertion of dactylus, two dorsodistal spiniform setae and four dorsal simple setae. Dactylus and unguis combined 1.3 times as long as propodus. Dactylus with three short setae along ventral margin and one sub-proximal seta on dorsal margin. Unguis third length of dactylus.

*Pereopod 2* (Fig. 9B): Basis 5.2 times as long as wide, with one ventrodistal simple seta on ventral margin and one dorsomedial broom seta. Ischium compact, with one ventrodistal simple seta. Merus half as long as basis, 3.2 times as long as wide, with two ventral simple setae. Carpus slightly shorter than merus, with five ventral simple setae and four dorsal simple setae. Propodus as long as carpus, with three spiniform setae and one distal simple seta on ventral margin and three simple setae and one broom seta on dorsal margin. Dactylus and unguis combined 1.2 times as long as propodus. Dactylus similar to that of female. Unguis half as long as dactylus.

*Pereopod 3* (Fig. 9C): Basis shorter than that of pereopod 2, with two broom setae each on dorsal and ventral margin and one ventrodistal simple seta. Ischium similar to that of pereopod 2. Merus 0.4 times as long as basis, with three ventral simple setae. Carpus as long as merus, with four ventral simple setae and three dorsal simple setae. Propodus slightly shorter than carpus, with two ventral spiniform setae, one ventrodistal simple seta and two dorsodistal simple setae and one dorsal broom seta. Dactylus and unguis combined 1.3 times as long as propodus. Unguis 0.3 times as long as dactylus.

*Pereopod 4* (Fig. 9D, E): Basis almost as long as that of pereopod 2, 5.9 times as long as wide, with one ventrodistal simple seta and one ventromedial broom seta. Ischium similar to that of pereopod 3. Merus 0.2 times as long as basis, with two ventrodistal simple setae. Carpus 1.7 times as long as merus, three times as long as wide, with two ventromedial simple setae and three distal simple setae. Propodus 0.8 times as long as carpus, 2.8 times as long as wide, with one ventromedial spiniform seta and eleven distal serrated spiniform setae. Dactylus and unguis combined 1.3 times as long as propodus. Unguis half as long as dactylus.

*Pereopod 5* (Fig. 9F, G): Basis longer than that of pereopod 4, 5.8 times as long as wide, with one ventrodistal simple seta and one dorsomedial broom seta. Ischium similar to that of pereopod 4. Merus 0.2 times as long as basis, twice as long as wide, with two distal simple setae. Carpus 1.4 times as long as merus, 3.6 times as long as wide, with two distal simple setae. Propodus slightly shorter than carpus, 3.6 times as long as wide, with four distal simple setae. Dactylus and unguis combined 1.1 times as long as propodus. Unguis half as long as dactylus.

*Pereopod 6* (Fig. 9H): Basis shorter than that of pereopod 5, 6.4 times as long as wide, with five broom setae and one ventrodistal simple seta. Merus 0.3 times as long as basis, with two ventral and two dorsal setae distally. Carpus 1.3 times as long as merus, 3.1 times as long as wide, with one ventral and one dorsal seta distally. Propodus subequal length of carpus, 4.3 times as long as wide, with three distal simple setae. Dactylus and unguis combined as long as propodus. Unguis half as long as dactylus.

*Pleopod* (Fig. 5E): Similar to that of female.

*Uropod* (Fig. 6G): Basal article 1.7 times as long as wide, with simple seta on distal margin. Exopod 3-articulate, article 3 twice as long as articles 1 and 2 combined, with two plumose setae on tip of article 3. Endopod 10-articulate, distal article with nine simple setae.

##### Remarks.

To check the morphological variation with size, ten specimens (1.64–2.73 mm) were partly dissected and the cheliped, the pereopod 1 and a ventral hyposphenium of pleonite were examined for different sizes and some variations were found: 1) in the cheliped, the dactylus and fixed finger of the males were modified in size: in the cheliped of the large sized males (over 2 mm), the cutting edge of the dactylus is extended and strongly curved and the processes on the proximal margin are prominently developed; 2) the number and size of setae on the pereopod 1 propodus increased with size; 3) a ventral hyposphenium of the male pleonite is modified from a rounded shape to a subrectangular shape, with body length reaching over 2 mm.

## Discussion

Sexual dimorphism within the genera *Ansphyrapus* and *Sphyrapoides* affects only their chelipeds, while in *Sphyrapus* and *Poligarida*, it affects their antennule, cheliped and pereopod 1. However, *Wandogarida canalicula* gen. n., sp. n. exhibits a greater level of sexual dimorphism: 1) the female body is wider than males, 2) in the antennule, the distal surface of the article 1 is round and naked in females, while that of the male has a vertical row of rough denticles and a deep groove; the outer flagellum of males is 5-articulate and the first and second articles have fringes of aesthetascs, but that of the female is 3-articulate and bears only one aesthetasc each on the first and second articles, 3) in the mandible, the incisor and lacinia mobilis of the left mandible of the female have distal denticles, but those of the male are distally pointed and naked and the setae of the setal row are shorter and more slender than those of the female. The setal row of the right mandible also differs (a row of five denticles and a small pointed seta in the male vs. a robust tripartite seta and three setulose setae in the female), 4) in the maxillule, the distal setae on the outer and inner endites of males are shorter than those of females, 5) the setae on the maxilla of males are diminished in size, as compared to those of the female, 6) the maxilliped endite of the male lacks inner setae and the distal margin has six naked and reduced spiniform setae, while that of the female has four plumose setae along the inner margin and eight distal spiniform setae, 7) in the cheliped, the basis, fixed finger and dactylus show dimorphism: the basis of the male is more robust than in the female, the cutting edge of the fixed finger has slender and pointed denticles and processes, but that of the female has distally broad denticles; the dactylus of the male has several denticles and processes along the cutting edge, but that of the female is naked and 8) the pereopod 1 of the male is longer than that of the female and the dactylus also shows a different shape in the both sexes: the ventral margin has small denticles in the male but spines in the female.

These results in the sexual dimorphism and morphological variation with body size shown in *W. canalicula* can be used as an important tool to divide easily and precisely males and females of the sphyrapodid species and upgrade our understanding how their life cycle or morphological function adapts to diverse environments.

## Supplementary Material

XML Treatment for Wandogarida

XML Treatment for Wandogarida canalicula
